# Dissecting the Genetic Basis Underlying Combining Ability of Plant Height Related Traits in Maize

**DOI:** 10.3389/fpls.2018.01117

**Published:** 2018-08-02

**Authors:** Zhiqiang Zhou, Chaoshu Zhang, Xiaohuan Lu, Liwei Wang, Zhuanfang Hao, Mingshun Li, Degui Zhang, Hongjun Yong, Hanyong Zhu, Jianfeng Weng, Xinhai Li

**Affiliations:** ^1^National Engineering Laboratory for Crop Molecular Breeding, Institute of Crop Science, Chinese Academy of Agricultural Sciences, Beijing, China; ^2^Institute of Cereal and Oil Crops, Hebei Academy of Agriculture and Forestry Sciences, Shijiazhuang, China; ^3^Yunnan Wenshanzhou Academy of Agricultural Sciences, Wenshan, China

**Keywords:** maize, plant height, combining ability, hybrid performance, QTL

## Abstract

Maize plant height related traits including plant height, ear height, and internode number are tightly linked with biomass, planting density, and grain yield in the field. Previous studies have focused on understanding the genetic basis of plant architecture traits *per se*, but the genetic basis of combining ability remains poorly understood. In this study, 328 recombinant inbred lines were inter-group crossed with two testers to produce 656 hybrids using the North Carolina II mating design. Both of the parental lines and hybrids were evaluated in two summer maize-growing regions of China in 2015 and 2016. QTL mapping highlighted that 7 out of 16 QTL detected for RILs *per se* could be simultaneously detected for general combining ability (GCA) effects, suggesting that GCA effects and the traits were genetically controlled by different sets of loci. Among the 35 QTL identified for hybrid performance, 57.1% and 28.5% QTL overlapped with additive/GCA and non-additive/SCA effects, suggesting that the small percentage of hybrid variance due to SCA effects in our design. Two QTL hotspots, located on chromosomes 5 and 10 and including the *qPH5-1* and *qPH10* loci, were validated for plant height related traits by Ye478 derivatives. Notably, the *qPH5-1* locus could simultaneously affect the RILs *per se* and GCA effects while the *qPH10*, a major QTL (PVE > 10%) with pleiotropic effects, only affected the GCA effects. These results provide evidence that more attention should be focused on loci that influence combining ability directly in maize hybrid breeding.

## Introduction

Maize (*Zea mays* L.), one of the most important crops worldwide, serves as food, animal feed, and is a new material for bioenergy production. Improving maize yield depends primarily on the application of hybrids, especially single-cross hybrids. To breed ideal hybrids with the highest grain yield, heterosis and combining ability (CA) of available germplasm have been implemented. The higher vigor of hybrids compared to their parents was defined as “heterosis" and was first observed by Darwin (1876), and then described in maize by East (1908) and Shull (1908). In order to widely utilize heterosis in hybrid breeding, a genetic mating scheme is usually used to identify elite parental lines and hybrid performance by analyzing general combining ability (GCA), and specific combining ability (SCA), respectively (Sprague and Tatum, 1942; Joshi et al., 2004). Analysis of the combining ability of sets of inbred lines play an important role in maize breeding programs for inbred line selection, heterotic group classification, heterotic pattern identification, and hybrid development (Zhang X.C. et al., 2017). Therefore, a better understanding of the genetic basis of combining ability can provide guidance for using them more effectively in maize improvement programs and hybrid performance prediction.

Combining ability has been applied extensively in hybrid breeding programs and has been commonly estimated using a diallel cross mating design (Griffing, 1956; Joshi et al., 2004; Liu et al., 2004; Basbag et al., 2007; Shukla and Pandey, 2008). GCA reflects additive allelic effects only and does not include dominance and epistasis. SCA, which only involves dominant and epistatic gene effects, indicates the degree to which hybrid performance deviates from the parents (Reif et al., 2007). Recently, development of next-generation sequencing technologies have allowed researchers to dissect the genetic basis of combining ability effects by linkage analysis with high density genetic maps (Giraud et al., 2017). Using three testcross populations and a backcross recombination inbred line (BCRIL), Qu et al. (2012) showed that the characteristics of the QTL for combining ability effects were similar to those of the QTL for BCRIL performance in rice. Combining linkage analyses with an NCII mating design, two major genes – *Ghd7* (Xue et al., 2008) and *OsPRR37* (Koo et al., 2013) – were confirmed to affect the GCA effects of heading date, spikelet per panicle, and plant height in rice (Liu C. et al., 2015). These results indicated that, as for trait *per se*, molecular markers could be used to dissect the genetic basis of GCA and SCA effects.

In China, breeding practice had shown that temperate maize germplasm can be divided into three main heterotic groups – A, B, and D. Group A includes subgroups PA (Partner A) and BSSS (including Reid); group B contains PB (Partner B) and Lan (Lancaster Sure crop); and group D consists of SPT (Sipingtou) and LRC (Lvda Red Cob) (Liu C.L. et al., 2015; Zhang X.C. et al., 2017). Genome-wide SNP markers showed that the genetic distances between the five subgroups were broad which could provide a potential pool of pyramiding favorable alleles for the improvement of inbred lines and varieties (Lu et al., 2009). Moreover, using an association mapping method on testcross data between 288 inbred lines and three testers, a strong relationship between GCA and SCA effects with population structure and genetic distance was identified (Larièpe et al., 2016). To enhance the power of CA effects QTL detection, elite inbred lines were frequently used to cross with another inbred lines which were chosen from opposite heterotic groups and defined as tester. For example, Qi et al. (2013) used four testers which belong to different heterotic groups to cross with 75 introgression lines to identify 56 significant QTL of GCA and SCA effects for five yield-related traits. Riedelsheimer et al. (2012) crossed 285 diverse dent inbred lines with two flint testers and predicted their combining abilities for seven biomass- and bioenergy-related traits using SNP and metabolite markers. Though only two testers were chosen, the prediction accuracies ranged from 0.72 to 0.81 for SNPs and from 0.60 to 0.80 for metabolites with whole-genome and metabolic prediction models. Therefore, uncovering the underlying genetic basis of combining ability will improve the selection of elite parental lines in hybrid breeding, and will increase the understanding of heterosis.

Maize grain yield is a quantitative trait with a complex genetic network, and its relatively low heritability makes it difficult to detect stable QTL across environments (Messmer et al., 2009). Compared with grain yield, yield-related traits such as plant height (PH), ear height (EH), and internode number (IN), which are the main components in maize plant architecture, frequently show high heritability and strong heterosis (Peiffer et al., 2014; Zhou et al., 2016; Li et al., 2017a). In general, ideal plant architecture can be used to directly determine the biomass, planting density, and grain yield. In order to allow sunlight to penetrate into the above ground canopy, previous studies have emphasized selection for optimal planting density by gradually increasing leaf angle, leaf area index, and leaf number (Stöckle and Kemanian, 2009; Pan et al., 2017). However, it is difficult to measure traits like leaf angle or leaf area index. Compared with these complex traits, plant and ear height are easily used to obtain accurate phenotypic data and suitable for genetic research. To date, using different mapping populations, hundreds of QTL for maize plant architecture traits have been identified^1^. While these advances are useful to understand the genetic basis and regulatory network of plant architecture traits *per se*, their contribution to combining ability, the main index for hybrid performance, are largely unknown.

In this study, a classic NCII mating design was used to produce two testcross populations by mating two tester lines with 328 Ye478 × Qi319 RILs. We performed QTL analysis and identified genetic loci underlying CA effects in these two testcross populations for maize plant height related traits. The objectives of the present study were to: (i) assess the correlation between RILs *per se*, combining ability, and hybrid performance; (ii) determine the genetic basis of combining ability and how it contributed to hybrid performance in maize; and (iii) validate the two hotspot QTL for RILs *per se* and GCA effects using Ye478 derivatives.

## Materials and Methods

### Genetic Materials

To detect the genetic basis of RILs *per se*, two elite inbred lines, Ye478 (as female) and Qi319, were crossed to produce 365 recombinant inbred lines (RILs). Ye478 and Qi319 were selected from PA and PB heterotic groups, respectively, and they were largely differentiated by both molecular and agronomic characteristics (Zhou et al., 2016; Zhang C.S. et al., 2017). In order to understand the genetic basis of combining ability, an NCII mating design was implemented (Griffing, 1956). Based on NCII design, two tester lines, Chang7-2 and Mo17, were selected as females to cross with the 365 RILs to generate the testcross population. Chang7-2, belonging to the SPT group, is a high GCA line derived from Huangzao4 that is widely used in the summer maize growing region of the Yellow and Huai Rivers. Mo17, an elite inbred line developed from Lan group, has been used extensively in commercial hybrid production. Due to the variation of flowering time for these lines, 328 RILs were successfully crossed both with Chang7-2 and Mo17 to produce 656 experimental hybrids. The testcross population which was produced by crossing the 328 RILs with Chang7-2 was defined as TC while crossing with Mo17 was defined as TM. Another testcross population, including 13 Qi319’s sister lines which were derived from the Pioneer commercial hybrid “PH78599,” 10 lines derived from Ye478, and the two parental lines Ye478 and Qi319, were also crossed with Chang7-2 and Mo17 to produce 50 tested hybrids and validate the two hotspot QTL for RILs *per se* and GCA in breeding. Detailed information for these parental lines is shown in **Supplementary Table S1**. The parents of the RILs, the four F_1_ hybrids that combined Ye478 and Qi319 with the two testers, were used as controls.

### Phenotypic Evaluation

All genetic materials including parental lines and hybrids were evaluated in four different environments (two locations in 2015 and two in 2016) in the summer maize-growing region of China, the 50 tested hybrids were only evaluated in 2016. The parental lines and hybrids were evaluated in the field in separate but adjacent experiments in an alpha lattice design with two replicates. Each plot consisted of two rows with 15 plants per row. The distance between plants in each plot was about 28 cm at a density of 60,000 plants/ha. The distance between plots was 60 cm. The field was managed according to normal agricultural practice. The middle ten plants in the central row of each plot were used for data collection. The three plant height related traits investigated were plant height (PH; in cm), ear height (EH; in cm), and internode number (IN). The methods for measuring these traits have been described in a previous study (Zhou et al., 2016). Phenotypic data for PH, EH, and IN were determined as the mean of measurements from ten individuals per plot.

### Phenotypic Data Analysis

To accurately measure phenotypic variations, we first corrected the raw phenotypic data by best linear unbiased estimations (BLUE) with the “*lme*” function in the R package^2^ “*lme4*”. The formulae as: Pheno ∼ 1 + Line + (1|Env) + (1|Rep) + (1| Line: Env), where Pheno is trait data; Line refers to inbred lines or hybrids and considers as fixed effects; Env indicates all environments, and Rep refers to the replications in each environment. The parentheses indicate random effects. The model matrix and grouping factors are separated by the vertical bar character“| ”. The interaction between factors are shown with “:”. Analyses including phenotypic distribution, correlation, and QTL were based on BLUE. The correlation between different trait datasets for three plant height related traits was assessed by the “*cor*” function in *R*.

The genetic variance effects of GCA and SCA effects were obtained in a joint linear mixed model analysis of both testcross populations over all four environments with *ASReml-R* package (Butler et al., 2007). The general model is:

$$\begin{array}{ll} Y_{ijklm}= & \mu +L_{i}+B_{j(i)}+GCA_{k}+GCA_{l}+SCA_{kl}+L\times \\ & GCA_{ik}+L\times GCA_{il}+L\times SCA_{ikl}+E_{ijklm}(\mathrm{model}\;1), \end{array}$$

where *Y_ijklm_* is the observation of the *m*th replication for a cross between the *k*th female and the *l*th male in the *j*th block and the *i*th location; *μ* indicates the overall mean; *L_i_* is the *i*th (*i* = 1 to *e*) fixed location (environment) effect. *B_j(i)_* indicates the *j*th (*j* = 1 to *b*) block within the *i*th location. GCA and SCA effects are assumed to be independently normally distributed (IND) with variances specific to each location. *GCA_k_*, and *GCA_l_* are the random GCA effects of the *k*th female and the *l*th male, respectively, IND following *N*(0, σ^2^_*GCA*_), *k*, *l* = 1 to *p* and *k* < *l*; *SCA_kl_* is the random SCA effects of the *k*th and the *l*th parents, IND following (0, σ^2^_*SCA*_) (*k*≠ *l*); *L^∗^GCA_ik_* and *L^∗^GCA_il_* indicates the random GCA by location interaction effect, IND following (0, σ^2^_*L*^∗^*G*_); *L^∗^SCA_ikl_* is the random SCA by location interaction effect, IND following (0, σ^2^_*L*^∗^*S*_); and *E_ijklm_* is the random error, and IND following (0, σ^2^_*E*_). The broad sense heritability (*H*^2^) of three plant architecture traits in the testcross population for combined environments were estimated using the formula (Knapp et al., 1985): *H*^2^ = σ^2^_*G*_/(σ^2^_*G*_ + σ^2^_*GL*_/L + σ^2^_*E*_/L × R), where σ^2^_*G*_ is the genetic variance of the hybrids, computed as the sum of the GCA and SCA components; σ^2^_*GL*_ is the interaction between hybrids and environments, σ^2^_*E*_ is the error variance, *L* is the number of environments, and *R* is the number of replications per location.

### Linkage Map and QTL Analysis

An ultra-high density linkage map of the RIL population was built by Zhou et al. (2016). In this study, 365 F_11_ RILs were genotyped using genotyping by sequencing (GBS) technology on an Illumina 2500 platform. This linkage map was constructed with 4602 bin markers and covered a total of 1533.72 cM of the ten maize chromosomes with an average distance of 0.33 cM between adjacent markers.

The QTL analysis was performed separately for the RILs *per se*, hybrid performance, GCA and SCA effects. The effects of GCA and SCA were obtained from model 1. In order to detect the genetic basis of combining ability, three datasets consisting of SCA/Chang7-2 (SC), SCA/Mo17 (SM), and GCA datasets were applied for the testcross populations. SC and SM represent the SCA effects which derived from the TC and TM testcross populations, respectively. Analysis of the QTL was conducted for each dataset by the composite-interval mapping (CIM) method using the *R/qtl* package (Broman et al., 2003). The 95% logarithm of the odds (LOD) values for the three traits across five datasets were obtained through 1,000 permutations. Due to the number of materials in different datasets is inconsistent, the LOD thresholds ranged from 2.97 to 3.56, with a mean value of 3. So the LOD score beyond 3.0 was defined as one QTL. The QTL confidence interval spanned the genomic regions corresponding to 1.5-LOD drop from the peak. The “*fitqtl*” function in the *R/qtl* package was applied to determine the proportion of phenotype variation explained by each QTL. QTL that were consistently detected for different traits in one given population or dataset were considered pleiotropic QTL if their estimated map position was within a distance of 20 cM, which is a common approach in comparative mapping for biparental populations (Frascaroli et al., 2007). However, for different datasets, the coincidence QTL indicates that the allelic substitution effect is consistent but in different conditions.

### Genomic Regions Transmitted From Ye478 to Its Descendants

Ye478 is a widely used foundation parent in China and most favorable alleles for plant height related traits from Ye478 can be transmitted to its derived lines (Liu et al., 2016). We analyzed the transmission of significant genomic regions (SGR) in the Ye478 derived lines using the maize SNP50 BeadChip on the Illumina Infinium platform from 41,101 SNPs (Weng et al., 2011). According to the genetic components analysis conducted by Liu C.L. et al. (2015), SGRs were defined by: (1) each derivative was compared with Ye478 using SNPs without missing genotypes in the Ye478 and with a missing rate less than 0.2 among its derivatives; and (2) a sliding window of 50 SNPs in steps of one SNP was used to calculate the percentage of genetic components that were the same as those of Ye478. If this percentage in each window was greater than 70%, then this region was defined as a significant genomic region from Ye478. Because semi-dwarfism was the typical trait of Ye478, only the genomic regions that included the two hotspots identified in the RILs for PH were analyzed. To test the statistical associations between phenotype and genotype in these two hotspots between Qi319’s sister lines and Ye478 derived lines, a total of 244 and 227 SNPs located on these two regions were used to test the significance of the genetic variations with a *t*-test.

## Results

### Performance of Traits in the RIL and Testcross Populations

**FIGURE 1:**
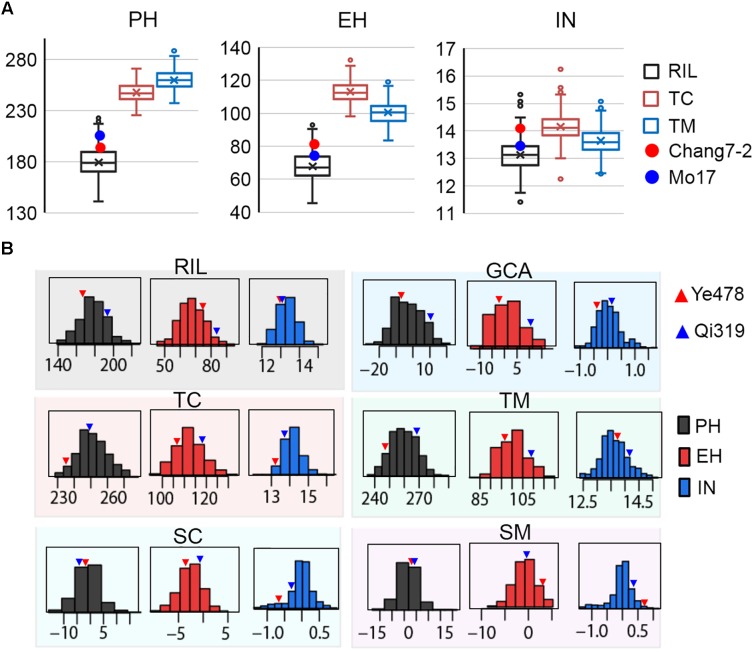
Extensive phenotypic variation of plant height related traits in different populations. **(A)** The means and ranges of three plant height related traits measured in the RILs and their testcross progenies. The red and blue spots indicate the tester lines Chang7-2 and Mo17, respectively. **(B)** The phenotypic variation of three traits in the six datasets. The histograms on each box indicate the phenotypic distribution of each trait dataset. The red and blue triangles indicate the parental lines Ye478 and Qi319, respectively. TC, the Chang7–2 testcross population; TM, the Mo17 testcross population; RIL, the recombinant inbred line population. GCA, general combining ability of RILs; SC and SM indicate the SCA effects of TC and TM, respectively. PH, plant height; EH, ear height; IN, internode number.

The means and ranges of three plant height related traits measured in the RILs and their testcross progenies were shown in **Figure 1A**. The average performance of testcross progenies was 238.71 cm (TC) and 255.42 cm (TM) for PH, 111.32 cm (TC), and 102.13 cm (TM) for EH, 14.44 (TC) and 13.41 (TM) for IN; all of these values were significantly higher than the corresponding values in the RIL population (**Figure 1A**). Notably, the percentages of testcross progenies that were significantly higher than paternal parents for IN were only 42.68% for TC and 9.14% for TM while those for PH and EH were approximately 100%. This result indicated that apparent heterosis for PH and EH was observed in the two testcross populations. In addition, with respect to testcross progenies, the average performances of TC were significantly higher than TM for both EH and IN (*P* < 0.01), which is consistent with the observation of parental lines Chang7-2 and Mo17 (**Figure 1A**). In the trial of testcross progenies, the Chang7-2/Mo17 × Qi319 controls were superior to the Chang7-2/Mo17 × Ye478 controls for all the three traits (**Figure 1B**). This result indicated that apparent higher heterosis of SPT/Lan × PA than heterosis of SPT/Lan × PB. Despite there is large difference between the controls of plant height related traits, some testcross progenies were less than the low value Chang7-2/Mo17 × Ye478, while some testcross progenies were more than the high value Chang7-2/Mo17 × Qi319, suggesting that the range in hybrids performance markedly transgressed the two controls. The values of the three traits in different populations and different datasets varied widely and showed a continuous and normal distribution (**Figure 1B**), indicating the presence of complex underlying genetic mechanisms for RILs *per se*, hybrid performance, and combining ability.

### Relationship Between Trait Values in Different Populations and Variance Analysis of Combining Ability

The correlations among the phenotypic values and GCA effects of RILs, the phenotypic values, and SCA effects of testcross populations for the three plant height related traits are shown in **Table 1**. Significant positive correlations were detected between the GCA effects and the RILs *per se* for all three traits (*r* > 0.55, *P* < 0.01). Similar results were observed for the relationship between the phenotypic values of hybrids and their paternal lines (*r* > 0.48, *P* < 0.01). Both GCA and SCA effects were significantly correlated with phenotypic values of all traits in the testcross populations, except the phenotypic values of hybrids for PH in the TM population (*r* = −0.08). This result indicated that both GCA and SCA play an important role in hybrid performance. However, the correlation coefficients among RILs *per se* and SCA effects were non-significant for PH and EH. These results suggested a higher genetic similarity between RILs *per se* and GCA effects rather than SCA effects.

**Table 1:** Phenotypic correlation (*r*) coefficients for plant height related traits in RILs and testcross populations.

	PH	EH	IN
RIL vs. GCA	0.63^∗∗^	0.77^∗∗^	0.55^∗∗^
RIL vs. SC	0.06	0.02	−0.21^∗∗^
RIL vs. SM	−0.03	−0.07	−0.22^∗∗^
TC vs. RIL	0.61^∗∗^	0.71^∗∗^	0.49^∗∗^
TC vs. GCA	0.93^∗∗^	0.93^∗∗^	0.89^∗∗^
TC vs. SC	0.17^∗^	0.34^∗∗^	−0.20^∗∗^
TM vs. RIL	0.58^∗∗^	0.72^∗∗^	0.48^∗∗^
TM vs. GCA	0.91^∗∗^	0.92^∗∗^	0.88^∗∗^
TM vs. SM	−0.08	0.25^∗∗^	−0.26^∗∗^
GCA vs. SC	−0.14^∗^	0.01	−0.48^∗∗^
GCA vs. SM	−0.29^∗∗^	−0.12^∗^	−0.55^∗∗^

*GCA denote the general combining ability of RIL lines; SC, SM indicate the special combining ability for hybrids of Chang7-2 × RILs, Mo17 × RILs. PH, plant height; EH, ear height; IN, internode number. ^∗^P < 0.05, ^∗∗^P < 0.01.*

Large and significant hybrid variances for all traits were observed in the testcross population, with transgressive segregation evident in the hybrids (**Figure 1**). The decomposition of the hybrid variance into GCA_k_ (RILs) and SCA_kl_ (tester × RILs) were found to be significant for all of the traits, indicating that both kinds of genetic effects were important in controlling the inheritance of the traits (**Table 2**). For all the traits except IN, the interaction between GCA_k_ and environments were both significant at *P* < 1%, whereas SCA_kl_ was not (except EH). Moreover, a higher σ^2^_GCA_/σ^2^_SCA_ ratio was observed in the present study, indicating that the predominance of additive gene action is more important for plant height related traits. Broad-sense heritabilities at the design level were high [between 0.82 (IN) and 0.89 (PH)] for all traits, suggesting that plant height related traits have a high transmitting ability to the next generation (**Table 2**).

**Table 2:** The combined analyses of variance for the plant height related traits in testcross population.

Source of variation	PH	EH	IN
GCA_k_	40.21^∗∗∗^	28.18^∗∗∗^	0.26^∗∗^
GCA_l_	9.87	8.66	0.21
SCA_kl_	11.82^∗∗∗^	5.66^∗∗∗^	0.11^∗^
L × GCA_k_	6.89^∗∗^	3.27^∗∗^	0.07
L × GCA_l_	2.79	0.36	0.05
L × SCA_kl_	1.42	2.09^∗∗^	0.07
Block	0.67	0.38	0.03
Residual	42.42	29.71	0.72
*H*^2^	0.89	0.88	0.82
GCA:SCA	4.24	6.50	4.27
%SCA	19.10	13.30	18.9

*^∗^P < 0.05, ^∗∗^P < 0.01, ^∗∗∗^P < 0.001. k and l indicated the kth male (RILs) and the lth female (Tester); PH, plant height; EH, ear height; IN, internode number*.

### QTL Detection

**FIGURE 2:**
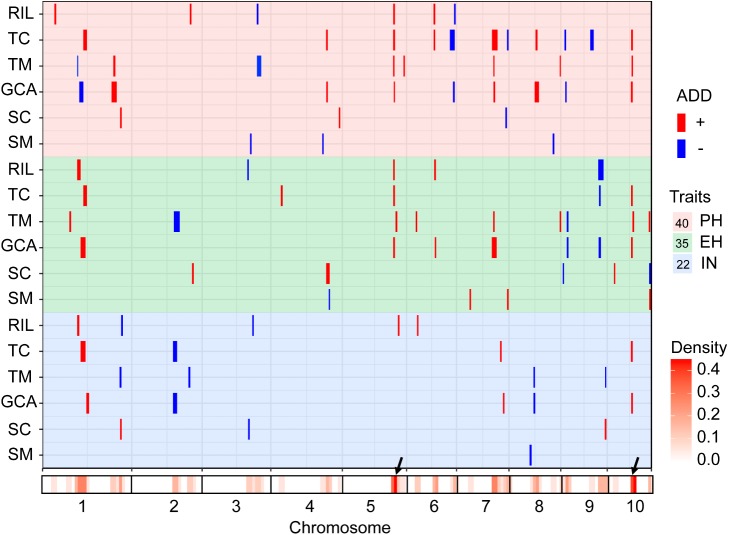
Chromosomal distribution of plant height related QTL identified in this study. QTL regions represented by the confidence interval for linkage mapping across the maize genome from the different datasets are shown as boxes. The width of the boxes shows the length of the confidence interval. The red and blue boxes indicate the QTL alleles with positive and negative values from Qi319 and Ye478, respectively. ADD: the additive effect value. PH, plant height; EH, ear height; IN, internode number. The *x*-axis indicates the genetic positions across the maize genome in Mb. The heatmap under the *x*-axis illustrates the density of plant height related QTL across the genome. The black arrows show the QTL hotspots. The trait dataset abbreviations match those in **Figure 1**.

**FIGURE 3:**
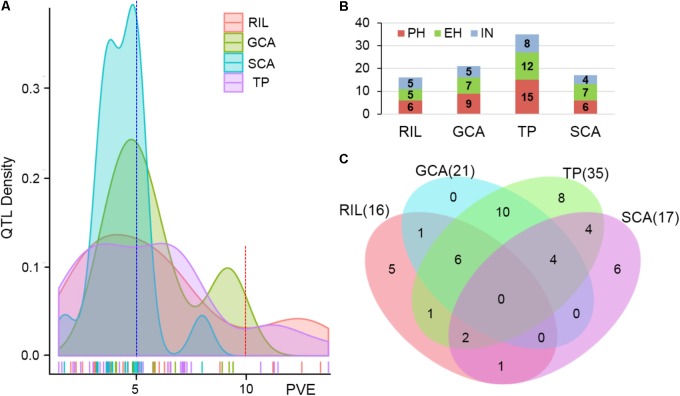
Characterization of QTL distribution associated with various datasets in maize. **(A)** Frequency distribution of QTL identified in RILs *per se*, hybrid performance, and combining ability effects based on the variance explained by each QTL. PVE, phenotypic variation explained. **(B)** QTL numbers distributed on each dataset for the three plant height related traits. PH, plant height; EH, ear height; IN, internode number. TP, testcross population. **(C)** Venn diagrams showing the number of QTL overlapping between RILs *per se* and GCA effects in the RIL population and hybrid performance and SCA effects combined Chang7-2 and Mo17 testcross population.

The testcross populations can be used to conduct QTL mapping for combining ability effects of the agronomic traits with NCII mating design. In total, 97 QTL were identified for the three traits evaluated for RILs *per se*, hybrid performance, GCA and SCA effects (**Figure 2**). Among these QTL, 40 were identified for PH, 35 for EH, and 22 for IN. These QTL were distributed across the 10 maize chromosomes and two QTL hotspots were identified on chromosomes 5 and 10 (**Figure 2**). The confidence intervals for these 97 QTL spanned physical distances from 1.40 to 31.70 Mb, with an average of 7.28 Mb compared to the B73 RefGen_v3 genome. The phenotypic variation explained by each QTL ranged from 1.02% to 14.15% of the variation and seven QTL located on chromosome 1, 3 and 10 for PH and EH explained more than 10% of the observed variation (**Figure 3A**). These results indicated that, as for RILs *per se* and hybrid performance, the genetic basis of combining ability especially GCA effects could be detected by QTL analysis.

### QTL for RILs *Per se* and GCA Effects

The QTL detected for the traits in the RIL population and for GCA effects are shown in **Figure 2** and **Table 3**. Thirty-seven QTL that affected the three traits in the two datasets were identified. Most of these QTL could individually explain less than 10% of the variation except for the QTL located on chromosomes 8 and 10 in GCA effects and IN (**Figure 3A** and **Table 3**). Fifteen QTL for PH were detected, including *qPH5-1* and *qPH6-2* loci simultaneously detected in RILs *per se* and in GCA effects (**Figure 3C**). At the *qPH5-1* locus, the Qi319 allele had a positive effect and increased PH by 15.09 cm compared with the Ye478 allele, and the locus raised PH GCA by 3.22. At the *qPH6-2* locus, the Qi319 allele had a negative effect. For EH and IN traits, a total of 12 and 10 QTL were identified, respectively. Four QTL, *qEH1-2*, *qEH5*, *qEH6-2*, and *qEH9-2*, which together explained 18.16 and 19.70% of the observed variation for EH and EH GCA effects were simultaneously detected in these two datasets (**Supplementary Figure S1**). For the four loci, the Qi319 allele could increase EH and EH GCA effects except *qEH9-2*. The two parental lines presenting both positive and negative allelic effects at the QTL leaded to observe the transgressive segregation in the hybrid progenies. Among these seven co-located QTL for these three traits in RILs and GCA effects, the direction of the parental contribution was identical, and this result is consistent with the significant positive correlation observed between the traits and GCA effects (**Figure 2** and **Table 1**). In addition, 9 and 14 QTL were characteristically detected in the RILs *per se* and GCA effects, respectively (**Figure 3B**). For example, the QTL cluster, *qPH10/qEH10-2/qIN10*, which had the largest phenotypic variation for PH and EH, was only stably detected on chromosome 10 in the GCA effects in the present study (**Supplementary Figure S2**). The results reflected the largely different genetic basis between RILs *per se* and GCA effects.

**Table 3:** QTL detected in RILs *per se* and GCA effects for three plant height related traits.

QTL	Chr	Marker interval	Interval (Mb)	RILs *per se*		GCA effects	
				*R*^2a^	A^b^	*R*^2a^	A^b^
*qPH1-1*	1	MK0188–MK0206	40.25–46.15	4.91	12.97		
*qPH1-2*	1	MK0330–MK0332	124.6–138.15			8.31	3.31
*qPH1-3*	1	MK0569–MK0617	234.15–251.45			2.69	-1.85
*qPH2*	2	MK1040–MK1052	197.05–202.50	3.96	11.37		
*qPH3*	3	MK1484–MK1549	168.00–187.45	9.90	-17.89		
*qPH4-1*	4	MK2000–MK2042	176.45–187.60			5.61	2.70
*qPH5-1*	5	MK2580–MK2601	173.10–178.60	7.12	15.09	8.00	3.22
*qPH6-1*	6	MK2880–MK2894	93.65–96.65	6.94	15.15		
*qPH6-2*	6	MK3432–MK3121	146.75–162.95	3.22	-10.42	3.55	-2.14
*qPH7-1*	7	MK3367–MK3384	125.00–130.45			5.42	2.65
*qPH8-1*	8	MK3699–MK3708	90.20–96.65			4.27	2.35
*qPH9-1*	9	MK4025–MK4049	13.45–18.25			1.02	-0.18
*qPH10*	10	MK4435–MK4445	82.00–84.95			11.22	3.86
*qEH1-2*	1	MK0331–MK0333	128.95–145.85	2.39	6.25	5.43	2.71
*qEH3*	3	MK1436–MK1458	153.55–158.65	6.23	-9.16		
*qEH5*	5	MK2579–MK2599	172.85–178.10	8.62	10.47	8.73	2.89
*qEH6-2*	6	MK2880–MK2903	93.65–99.10	6.05	8.67	3.98	1.96
*qEH7-2*	7	MK3367–MK3382	125.00–129.25			1.74	1.08
*qEH9-1*	9	MK4011–MK4080	11.05–25.90			2.68	-1.72
*qEH9-2*	9	MK4243–MK4265	129.60–135.05	1.10	-4.77	1.56	-1.00
*qEH10-2*	10	MK4433–MK4450	80.75–86.45			11.71	3.35
*qIN1-1*	1	MK0331–MK0333	128.95–145.85	3.90	0.44	2.93	0.15
*qIN1-2*	1	MK0666–MK0682	263.10–268.55	4.99	-0.50		
*qIN2-1*	2	MK0941–MK0943	129.95–153.65			5.86	-0.21
*qIN3*	3	MK1466–MK1512	161.60–175.40	12.30	-0.77		
*qIN5*	5	MK2628–MK2649	188.55–193.95	8.30	0.65		
*qIN6*	6	MK2815–MK2820	36.20–40.05	3.45	0.42		
*qIN7*	7	MK3434–MK3455	147.45–152.85			3.78	0.17
*qIN8*	8	MK3685–MK3698	83.30–89.35			4.53	-0.19
*qIN10*	10	MK4432–MK4450	80.35–86.45			4.24	0.18

*^a^R^2^ represents the phenotypic variation explained by each QTL. ^b^Additive effects are averages of the means of RILs harboring alleles from the Qi319 and the means of RILs possessing alleles of Ye478 at the significant loci. Positive values of the additive effect indicate that alleles from Qi319 were in the direction of increasing the trait score. The trait dataset abbreviations match those in **Figure 1**. PH, plant height; EH, ear height; IN, internode number.*

### QTL for Hybrid Performance and Non-additive/SCA Effects

To deepen our understanding of the genetic basis of heterosis, QTL that were identified for hybrid performance and non-additive/SCA effects in the TC and TM testcross populations were compared and 60 QTL were resolved (**Figure 3B** and **Supplementary Table S2**). The number of QTL identified in the two testcross populations for hybrid performance ranged from 4 to 11 with an average of 6.83 for these three traits, and most of these QTL explained less than 10% phenotypic variance (**Figures 3A,C**). A total of six QTL could be simultaneously detected in the two testcross populations and the direction of the parental contribution to these QTL was identical (**Supplementary Figure S1B**). However, all four QTL detected in the Mo17 testcross population for IN showed the negative effect (**Figure 2**). This result indicated that alleles from the Qi319/Mo17 heterozygote decreased maize internode number compared with the Ye478/Mo17 heterozygote. In addition, a total of nine QTL could be simultaneously detected in the RIL population for RILs *per se* (**Figure 3C**). This result is consistent with the significant positive correlation observed between the phenotypic values of hybrids and their paternal lines (**Table 1**). Notably, all of the QTL (except *qEH6-2*) identified in the GCA effects have significant effects in the testcross populations which means GCA contributes a lot to hybrid performance (**Figure 3C**).

In the present study, the SCA variation component was significant for all traits and the proportion of SCA represented about 17% of the genetic variance in hybrids (**Table 1**). The significant contribution of non-additivity to overall genetic variation for considering traits suggested that our experimental system is adaptive to understand the factors underlying non-additive inheritance by QTL analysis. A total of 19 QTL controlling the three plant height related traits showed non-additive effects. Among these QTL, two QTL could be simultaneously detected for EH with non-additive effects in TC and TM (**Supplementary Figure S1B**). However, most of these QTL explained less than 5% phenotypic variance suggesting that many loci controlling traits showed minor non-additive effects that were hardly detectable in our detection model (**Figure 3A**).

### Validation of the Two Hotspot QTL for RILs *Per se* and GCA by Derivatives

**FIGURE 4:**
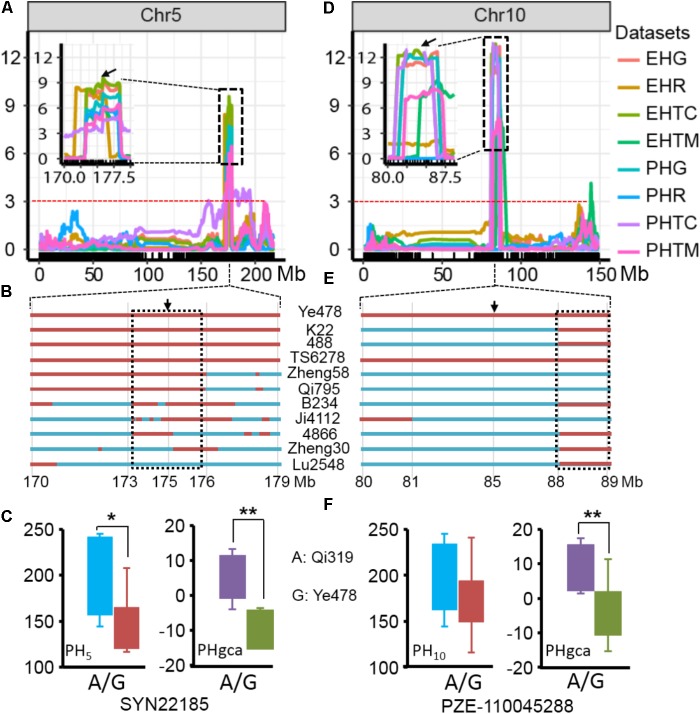
Validation of the two hotspot QTL for RILs *per se* and GCA. **(A,D)** The two hotspots located on chromosomes 5 and 10 identified by linkage analysis. The box shows a magnification of the QTL candidate interval region on chromosomes 5 and 10. The black arrows indicate the significant SNPs, which located in the QTL candidate interval region and detected by *t*-test between the Qi319’s sister lines and Ye478 derived lines for PH and PH GCA. The dashed horizontal line depicts the significance threshold [–Log10(*P)* = 3]. PHR and EHR indicate the plant height and ear height in the RIL population. PHG and EHG indicate the general combining ability of PH and EH, respectively. PHTC, PHTM, EHTC, and EHTM indicate the plant height and ear height in the TC and TM testcross population. **(B,E)** Distributions of SGR from Ye478 in its derivatives in the two hotspots on chromosomes 5 and 10, respectively. Red bars indicate the fragments or SNPs from Ye478, blue was chromatin from other lines. The black arrows indicate the significant SNP, which was detected by a *t*-test. The dashed box indicates the small SRG which could be detected in at least seven derivatives. **(C,F)** Boxplot of PH and PH GCA distribution at the peak SNP on chromosomes 5 and 10 for the lines representing the Qi319’s sister lines and Ye478 derived lines. Differences between the genotypes were analyzed using Student’s *t*-test. ^∗^*P* < 0.05, ^∗∗^*P* < 0.01.

The results of the present study showed the highly concentrated distribution of QTL in a few chromosomal regions. As shown in **Figure 2**, the boxes of the QTL across the maize genome revealed that these QTL were clustered according to the phylogenetic relationships of traits rather than distributed randomly on the chromosomes (**Figure 2**). Consistent with the significant correlation pattern of the traits *per se* (**Supplementary Table S3**), the QTL associated with PH, EH, and IN clustered as more closely linked in different combinations, with the expectation of two hotspots located on chromosome 5 and chromosome 10 as key roles in the regulation of plant architecture (**Supplementary Figure S2** and **Supplementary Table S4**). The *qPH5-1* locus, which was significantly associated with PH and EH in the RIL population, were all consistently followed by the significant effects on hybrid performance in the testcross and GCA effects (**Figure 4A**). However, the QTL hotspot on chromosome 10, which included the largest phenotype variation *qPH10* locus, were only simultaneously detected for these three traits in the testcross population and GCA effects (**Figure 4D**). The significant genomic regions (SGR) located on these two hotspots distributed in the 10 Ye478-derived lines are shown in **Figures 4B,E**. For the *qPH5-1* locus, the favorable alleles associated with PH were mainly transmitted from Ye478 to its derived lines except Lu2548 (**Figure 4B**). For *qPH10*, only TS6278 inherited all SGR associated with PH from Ye478, while the others only received a small number of SGR (**Figure 4E**). In addition, SNPs located on these two hotspots between Ye478 derived lines and Qi319’s sister lines were calculated using a *t*-test to estimate the effects on RILs *per se* and the GCA, and the results are shown in **Supplementary Figure S3**. Most importantly, the 13 lines directly selected from hybrid PH78599 possess the same Qi319 allele (AA) at the SYN22185 locus on chromosome 5 and had an average value of 188.35 cm and 3.62 for PH and PH GCA, which are significantly higher than the 10 lines that harbor the Ye478 allele (GG, *P* < 0.05, **Figure 4C**). However, the small SGR located on chromosome 10 did not contain the SNP PZE-110045288, which is significantly associated with PH GCA (*P* < 0.01), but not significantly associated with PH (*P* = 0.16, **Figure 4F**).

## Discussion

### Compared With RILs *Per se*, Combining Ability Is More Important for Hybrid Phenotypes

Prediction of hybrid performance is important in hybrid breeding. With the development of next generation sequencing technologies, heterosis or hybrid performance can be predicted by QTL which possibly provide targets for marker-assisted selection (MAS) in maize hybrid breeding (Cerna et al., 1997; Joshi et al., 2001; Jordan et al., 2003; Liu et al., 2004; Lariepe et al., 2012; Li et al., 2017b). In the present study, a total of 35 QTL were detected for hybrid performance in these two testcross populations and only six QTL (20.7%) were significantly affected the RILs *per se* (**Figure 3B**). For example, *qPH5-1* was concurrently detected in RILs *per se* and hybrid performance for PH and EH, whereas the QTL detected with hybrid performance on chromosome 10 appears not to be associated with inbred *per se* performance (**Figures 4D,F**). Loci commonly detected in hybrid performance and RILs *per se* may explain the higher correlation between the two progeny values observed for high heritability traits such as plant height related traits, and this result is consistent with a previous study (Austin et al., 2001). However, about 60% of the QTL were specific for RILs *per se* when compared with the QTL in testcross population (**Figure 3**). Similar results were obtained by Mihaljevic et al. (2005) and Peng et al. (2013), who found that about 40–75% QTL detected with inbreds *per se* for agronomic traits were not associated with those QTL detected with hybrid performance. In addition, among the 35 QTL detected for hybrid performance in these two testcross populations, only six QTL could be simultaneously identified in TC and TM (**Supplementary Figure S1B**). The 29 QTL detected in only one testcross population indicated that testers choice was a complex factor affecting the power of the QTL detection in the testcross population (Frascaroli et al., 2009). This finding is consistent with the facts that Chang7-2 and Mo17 belong to the different heterotic groups. QTL identification for inbreds *per se* performance is largely done to reflect additive effects, whereas QTL for hybrid performance in the testcross population is determined by the interaction effects between the population’s parental alleles with those of the testers (Schon et al., 2010). Because of the potential masking dominance effects in the testers, the QTL for which there is variation in the RILs could be identified in the testcross population when the testers offered the recessive alleles (Smith, 1986; Hallauer, 1990). Therefore, QTL studies estimating the performance of hybrids are needed to determine if the same or different QTL are identified for inbreds *per se* and hybrid performance.

The value of a hybrid is traditionally determined by two components: GCA and SCA effects (Sprague and Tatum, 1942). Most previous studies had found that increasing the prediction efficiency of hybrid performance for grain yield related traits mostly depended on the models enhancing the GCA approach with SCA estimate (Schrag et al., 2006; Giraud et al., 2017). Therefore, identification of the significant loci for hybrid values and their GCA and SCA components with DNA markers would improve the efficiency of hybrid prediction, and lead to accelerated understanding of the mechanism of heterosis. In the present study, Ye478 and Qi319 were selected from PA and PB heterotic groups, respectively, and they were largely differentiated by both molecular and agronomic characteristics (Zhou et al., 2016; Zhang C.S. et al., 2017). The two tester lines, Chang7-2 and Mo17, which belong to SPT and Lan heterotic groups were selected as females to cross with the RILs by NCII mating design. As shown in **Figure 1**, the Chang7-2/Mo17 × Qi319 controls were superior to the Chang7-2/Mo17 × Ye478 controls for all the traits in the testcross population. This result indicated that apparent higher heterosis of SPT/Lan × PA than heterosis of SPT/Lan × PB. In addition, the values of the three traits in different datasets varied widely and showed a continuous and normal distribution (**Figure 1B**), which indicated QTL mapping could be used to detect the genetic basis of combining ability. Compared with the 21 QTL identified in the GCA effects, all of the GCA QTL (except *qEH6-2*) have significant effects for hybrid performance which means GCA contributes heavily to hybrid performance (**Figure 3B**). However, only ten non-additive/SCA QTL overlapped with hybrid performance and 63.15% QTL contributing to plant height related traits produce minor non-additive effects (**Supplementary Figure S1A** and **Supplementary Table S2**). This result indicated that alleles with a large effect on plant height related traits may have been either fixed or purged during long-term artificial selection (Schnable and Springer, 2013). Hence, all above results show that hybrid performance is mostly affected by GCA loci; this result is consistent with GCA explaining over 70% of hybrid variation in the testcross population. Therefore, the combining ability loci identified in this study, especially for the GCA effects loci would be more useful for maize hybrid breeding.

### Important Considerations for Combining Ability Estimation

It is well known that classical diallel designs were applied to explain the genetic basis of combining ability (Griffing, 1956). Currently, with the development of DNA markers, the genetic basis of combining ability can be easily detected by linkage analysis with NCII mating design for different QTL mapping populations, such as RIL, double haploids (DH), F_2_, F_2:3_, and BC_1_ (Lv et al., 2012; Qu et al., 2012; Qi et al., 2013). However, populations like F_2_, F_2:3_, and BC_1_ usually segregate at the whole genome level and are heterozygous at most loci. If estimating GCA effects using the lines derived from these populations, both the complex genetic basis of tested individuals and the effects from testers and tested × testers interaction must be considered. Compared with these populations, RIL and DH populations are valuable materials with high levels of homozygosity which lead to a higher estimate of additive genetic variance, and dominance interaction could be eliminated. In addition, high density genetic linkage maps have been constructed with next generation sequencing and successfully used in the study of phenotypes such as hybrid vigor, which will also accelerate the genetic analysis of combining ability (Zhou et al., 2012; Huang et al., 2016).

In testcross populations, the performance of hybrids is largely determined by the additive variance (σ^2^_GCA_/σ^2^_SCA_ > 3.6), however, non-additive variance, which mainly comes from the allelic interactions between the tested lines and the testers, also contributed substantially to the observed variation in the hybrid progeny. This result is consistent with previous studies showing that SCA usually explained about 10% of the hybrid variation for the traits tested (Schrag et al., 2006, 2009, 2010; Fischer et al., 2008; Technow et al., 2014). Although the proportion of SCA effects is restricted compared with GCA, it might be sufficient to impede an accurate estimation of GCA effects especially only one or two of testers were chosen from the other groups (Reif et al., 2007). Thus, the choice of testers is a crucial issue that can affect the genetic variance of testcross progenies and the power of QTL detection. To enhance the power of GCA QTL mapping, a genetically broad-based tester could contribute less to the lines × testers interaction than testers with a narrow genetic basis (Matzinger, 1953). Recently, studies have shown that synthetic populations from different heterosis groups (such as Dent and Flint) may be more suitable as testers than inbred lines to accurately estimate the GCA effects (Giraud et al., 2014, 2017; Kadam et al., 2016). Firstly, a synthetic population generally has wide genetic variability which makes it is better than an inbred line to estimate the GCA effects. Secondly, the synthetic populations have various genotypes, which can eliminate the deviation of GCA effects caused by dominance, over-dominance, and epistasis. Thirdly, synthetic populations including a series of inbred testers can effectively reduce the number of combinations, which is not only cost saving, but also can acquired the precision of agronomic traits for controlling the experimental error (Lv et al., 2012).

### Combining Ability Shared the Different Genetic Basis With RILs *Per se*

Combining ability has been successfully applied in crop and livestock hybrid breeding to evaluate parental performance for more than 70 years (Ahangar et al., 2008; Moterle et al., 2011). Therefore, parental inbred lines with high combining ability are considered critical for parental line selection and for the development of superior hybrids (Duvick et al., 2004). In the present study, the performance of GCA effects was significantly correlated to the performance of RILs for all three plant height related traits (0.55 < *r* < 0.77, *P* < 0.01, **Table 1**). On the genetic basis, seven GCA QTL for plant height related traits were found to pass their effects from parents to hybrid progeny. However, several QTL, such as *qPH10* and *qEH10-2*, had larger effects in GCA effects but had small effects in the RIL population, while other QTL had minor effects for RILs *per se* and could hardly be detected in GCA effects (**Table 3**). In addition, traits with low heritability, such as yield per plant, 100-kernel weight, and kernel number per row usually showed weak correlations between the performance of inbred lines and their GCA effects in a previous report (Lv et al., 2012; Qi et al., 2013). These results reflected the largely different genetic basis between RILs *per se* and combining ability especially GCA effects. Therefore, GCA and traits *per se* for a given inbred line are occasionally not improved synchronously. For example, Nongda 108, an excellent commercial hybrid in China, is a combination of Huang C and X178, two inbred lines with high yields and other desirable traits. However, no other commercial hybrid has been created using these two lines as parents, indicating that these lines do not possess a high GCA, but only a specific combining ability. The high yield of the two lines indicated that yield-related loci are distinctly improved in breeding, while the low GCA effects suggested that genetic loci for GCA effects were not modified along with the improvement of yield-related loci (Lv et al., 2012).

### Potential Utilization of Two QTL Hotspots in Maize Hybrid Breeding

The development of single cross hybrids is largely dependent on elite maize inbred lines. Therefore, selecting foundation parents with high GCA effects is crucial in maize breeding (Rojas and Sprague, 1952; Li and Wang, 2010). However, evaluation of the combining ability with traditional methods is labor intensive and time-consuming because vast hybrid combinations are required. Marker-assisted selection offers a viable and cost-effective way to improve complicated agricultural traits with significant QTL (Yousef and Juvik, 2001; Eathington et al., 2007; Qi et al., 2013). An interesting result of this study is the highly concentrated distribution of QTL in a few chromosomal regions, which indicated the presence of QTL hotspots which could be utilized with MAS (**Figure 2**). These findings are particularly true for the region surrounding the MK2575–MK2601 locus on chromosome 5 and the MK4435–MK4464 locus on chromosome 10, where the pleiotropic QTL for at least two traits were detected in different combinations (**Supplementary Figure S2**). Moreover, similar concentrated distributions of QTL have also been observed in previous studies for plant architecture and yield-related traits (Qi et al., 2013; Zhou et al., 2016; Li et al., 2017a; Zhang C.S. et al., 2017). For example, Weng et al. (2011) detected a dwarf locus in maize bin 5.05–5.06, which was consistent with the present results regarding *qPH5-1*. We observed that *qPH5* was significantly associated with PH and PH GCA effects, and were highly conserved in at least seven Ye478 descendants, most of which have been widely used in Chinese maize hybrid breeding programs (**Figures 4A–C**). The performance of lines *per se* and the GCA effects for the Ye478-derived lines suggested that as a foundation parent the typical plant architecture of Ye478 had been transmitted to its derivatives with similar agronomic traits (Liu et al., 2016). The hotspot *qPH10* locus was only closely linked to a major QTL for PH GCA effects in the testcross population. Moreover, the 13 lines directly selected from hybrid PH78599 possessing the same Qi319 allele (AA) at the SNP PZE-110045288 for PH GCA effects is significantly higher than that of the lines that harbor the Ye478 allele (GG, *P* < 0.01), while no significant effects were detected for PH (*P* = 0.16, **Figure 4F**). As a result, this genomic region was not highly conserved in Ye478 descendants, suggesting that this may be a novel allele for plant architecture GCA in Ye478 (**Figure 4E**). Previous studies have shown that recurrent selection is a useful strategy to improve the performance of a population (Lv et al., 2012). This means that the favorable alleles for GCA effects in an advanced population could be accumulated with cycles of selection and phenotype improvement and might be due to the pyramiding of more favorable alleles. Therefore, the favorable alleles detected for traits *per se* or GCA effects in the present study may be useful in improving the performance of traits *per se* and GCA effects for inbred lines.

## Ethics Statement

The experiments comply with the ethical standards in the country in which they were performed.

## Author Contributions

ZZ performed the experiments and wrote the paper. CZ, XLu, LW, HY, ZH, ML, DZ, and HZ, performed the experiments and revised the paper. JW and XLi designed the experiments.

## Conflict of Interest Statement

The authors declare that the research was conducted in the absence of any commercial or financial relationships that could be construed as a potential conflict of interest.

## Footnotes

1 http://www.maizegdb.org

2 http://www.r-project.org/

## Supplementary Material

FIGURE S1 Characterization of QTL distribution associated with various datasets in maize. **(A)** Frequency distribution of QTL identified in six datasets based on the variance explained by each QTL. PVE, phenotypic variation explained. **(B)** Venn diagrams showing the number of QTL overlapped between SCA effects in Chang7-2 and Mo17 testcross population. **(C)** QTL numbers distributed on each dataset for the three plant height related traits. PH, plant height; EH, ear height; IN, internode number. The trait dataset abbreviations match those in **Figure 1**.

FIGURE S2 The information of QTL identified in different populations. **(A)** Chromosomal distribution of plant height related QTL identified in six datasets. QTL regions represented by the confidence interval for linkage mapping across the maize genome from the different datasets are shown as boxes. The *x*-axis indicates the genetic positions across the maize genome in Mb. The heatmap under the *x*-axis illustrates the density of plant height related QTL across the genome. PH, plant height; EH, ear height; IN, internode number. **(B)** Venn diagrams showing the number of QTL shared between the three plant height related traits in different datasets. The trait dataset abbreviations match those in **Figure 1**.

FIGURE S3 Scatter diagram showing the significant SNPs located in the two hotspots on chromosomes 5 and 10 for traits *per se*, hybrid performance, and GCA effects. **(A,B)** Significant SNPs located on chromosome 5. **(C,D)** Significant SNPs located on chromosome 10. The red arrow indicates the most significant SNP.

TABLE S1 Information of 27 maize inbred lines used in the present study.

TABLE S2 QTL associated with plant height related traits detected for the phenotypic performance in the testcross population and non-additive/SCA effects.

TABLE S3 Phenotypic correlation (*r*) coefficients for plant height related traits in different trait datasets.

TABLE S5 Pleiotropic QTL identified in different combinations for plant height related traits.
