# Supplementary figures and images for: Dissecting the Genetic Basis Underlying Combining Ability of Plant Height Related Traits in Maize
Source: Front Plant Sci. 2018 Aug 2;9:1117. doi: 10.3389/fpls.2018.01117 (PMC6083371; doi:10.3389/fpls.2018.01117)

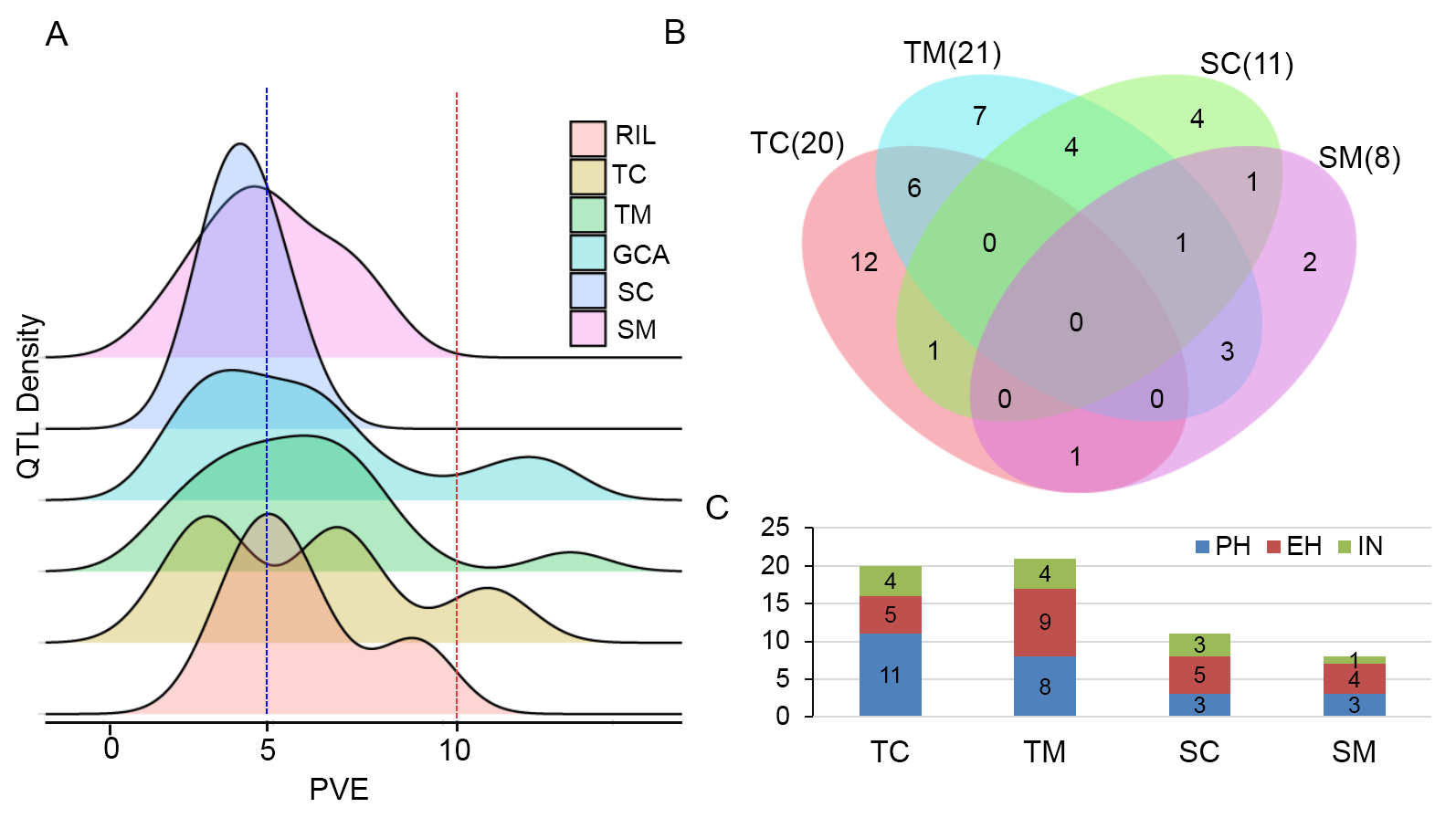

Supplement: FIGURE S1 — Characterization of QTL distribution associated with various datasets in maize. (A) Frequency distribution of QTL identified in six datasets based on the variance explained by each QTL. PVE, phenotypic variation explained. (B) Venn diagrams showing the number of QTL overlapped between SCA effects in Chang7-2 and Mo17 testcross population. (C) QTL numbers distributed on each dataset for the three plant height related traits. PH, plant height; EH, ear height; IN, internode number. The trait dataset abbreviations match those in Figure 1. [file Image_1.TIF]

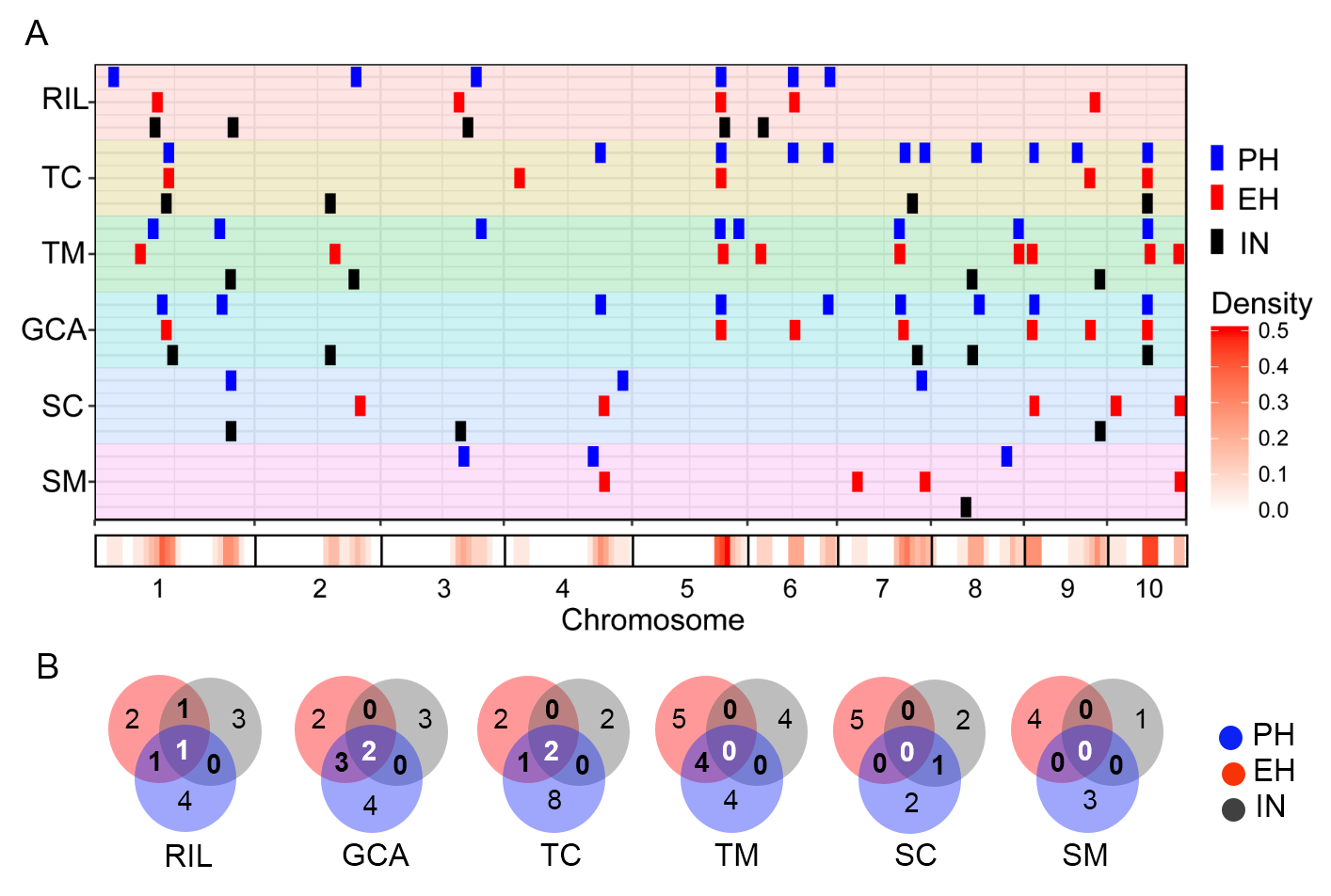

Supplement: FIGURE S2 — The information of QTL identified in different populations. (A) Chromosomal distribution of plant height related QTL identified in six datasets. QTL regions represented by the confidence interval for linkage mapping across the maize genome from the different datasets are shown as boxes. The x-axis indicates the genetic positions across the maize genome in Mb. The heatmap under the x-axis illustrates the density of plant height related QTL across the genome. PH, plant height; EH, ear height; IN, internode number. (B) Venn diagrams showing the number of QTL shared between the three plant height related traits in different datasets. The trait dataset abbreviations match those in Figure 1. [file Image_2.TIF]

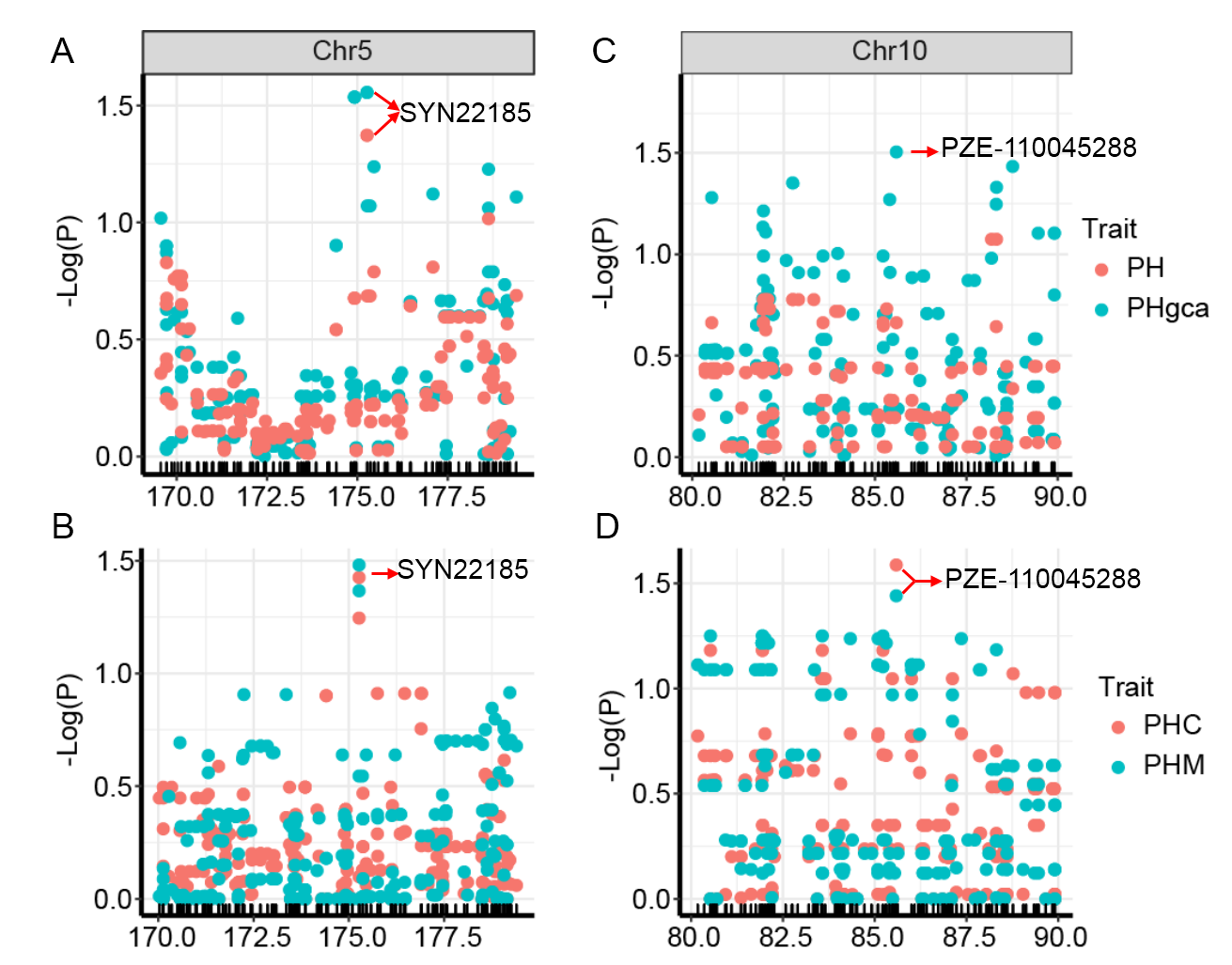

Supplement: FIGURE S3 — Scatter diagram showing the significant SNPs located in the two hotspots on chromosomes 5 and 10 for traits per se, hybrid performance, and GCA effects. (A,B) Significant SNPs located on chromosome 5. (C,D) Significant SNPs located on chromosome 10. The red arrow indicates the most significant SNP. [file Image_3.TIF]
