# Supplementary material for: Dissecting the Genetic Basis Underlying Combining Ability of Plant Height Related Traits in Maize
Source: Front Plant Sci. 2018 Aug 2;9:1117. doi: 10.3389/fpls.2018.01117 (PMC6083371; doi:10.3389/fpls.2018.01117)
Supplement: TABLE S1 — Information of 27 maize inbred lines used in the present study. [file Table_1.DOCX]

Table S1. Information of 27 maize inbred lines used for the present study.

| No. | Inbred Line | Pedigree | Heterotic group |
| --- | --- | --- | --- |
| N1 | Qi318 | American Pioneer Hybrid P78599 | PB |
| N2 | R18 | American Pioneer Hybrid P78599 | PB |
| N3 | 89-1 | American Pioneer Hybrid P78599 | PB |
| N4 | SH15 | American Pioneer Hybrid P78599 | PB |
| N5 | Jinhuang59 | American Pioneer Hybrid P78599 | PB |
| N6 | 31778 | American Pioneer Hybrid P78599 | PB |
| N7 | Jinhuang55 | American Pioneer Hybrid P78599 | PB |
| N8 | Qi319 | American Pioneer Hybrid P78599 | PB |
| N9 | Dan599 | American Pioneer Hybrid P78599 | PB |
| N10 | Duohuang29 | American Pioneer Hybrid P78599 | PB |
| N11 | Shen136 | American Pioneer Hybrid P78599 | PB |
| N12 | Nongda178 | American Pioneer Hybrid P78599 | PB |
| N13 | P138 | American Pioneer Hybrid P78599 | PB |
| N14 | Dan3130 | American Pioneer Hybrid P78599 | PB |
| N15 | Lu2548 | Qi205 × Ye478 | PA |
| N16 | Zheng30 | Zheng20×Ye478 | PA |
| N17 | B234 | 9046 × Ye478 | PA |
| N18 | Ji4112 | U8112 × B37HT | PA |
| N19 | K22 | Ye478 × K11 | PA |
| N20 | Zheng58 | Variant plants from Ye478 | PA |
| N21 | 488 | Ye478 × 478-31 | PA |
| N22 | 4866 | Tie7922 × Ye478 | PA |
| N23 | TS6278 | Nan602 × Ye478 | PA |
| N24 | Qing795 | Improved from Zheng58 | PA |
| N25 | Ye478 | U8112 × Shen5003 | PA |
| N26 | Mo17 | C103 × 187-2 | Lan |
| N27 | Chang7-2 | (HZ4 × Wei95) × S901 | SPT |

PA: Partner A;

PB: Partner B;

SPT: Sipingtou, a Chinese landrace and its derivatives;

Lan: Lancaster Sure Crop lines
