# Supplementary material for: Dissecting the Genetic Basis Underlying Combining Ability of Plant Height Related Traits in Maize
Source: Front Plant Sci. 2018 Aug 2;9:1117. doi: 10.3389/fpls.2018.01117 (PMC6083371; doi:10.3389/fpls.2018.01117)
Supplement: TABLE S2 — QTL associated with plant height related traits detected for the phenotypic performance in the testcross population and non-additive/SCA effects. [file Table_2.DOCX]

Table S2 QTL associated with plant height related traits detected for the hybrid performance in the testcross population and SCA effect.

| QTL | Chr | Interval (Mb) | TC | | TM | | SC | | SM | |
| --- | --- | --- | --- | --- | --- | --- | --- | --- | --- | --- |
|  |  |  | R^2a^ | A^b^ | R^2a^ | A^b^ | R^2a^ | A^b^ | R^2a^ | A^b^ |
| *qPH1-2* | 1 | 124.6-138.15 | 7.34 | 9.11 | 8.30 | 8.96 |  |  |  |  |
| *qPH1-3* | 1 | 234.15-251.45 |  |  | 5.57 | -7.23 | 5.11 | 2.07 |  |  |
| *qPH3* | 3 | 168.00-187.45 |  |  | 1.93 | -4.32 |  |  | 4.59 | -1.84 |
| *qPH4-1* | 4 | 176.45-187.60 | 8.80 | 10.01 |  |  |  |  | 8.05 | -2.43 |
| *qPH4-2* | 4 | 231.75-235.45 |  |  |  |  | 5.13 | 2.11 |  |  |
| *qPH5-1* | 5 | 173.10-178.60 | 5.51 | 8.02 | 5.78 | 7.73 |  |  |  |  |
| *qPH5-2* | 5 | 208.50-212.35 |  |  | 2.63 | 5.24 |  |  |  |  |
| *qPH6-1* | 6 | 93.65-96.65 | 3.31 | 6.14 |  |  |  |  |  |  |
| *qPH6-2* | 6 | 146.75-162.95 | 3.41 | -6.25 |  |  |  |  |  |  |
| *qPH7-1* | 7 | 125.00-130.45 | 0.50 | 2.82 | 6.26 | 7.71 |  |  |  |  |
| *qPH7-2* | 7 | 165.55-170.60 | 2.98 | -5.94 |  |  | 3.29 | -1.68 |  |  |
| *qPH8-1* | 8 | 90.20-96.65 | 2.48 | 5.36 |  |  |  |  |  |  |
| *qPH8-2* | 8 | 148.15-154.00 |  |  | 3.03 | 5.31 |  |  | 4.96 | -1.86 |
| *qPH9-1* | 9 | 13.45-18.25 | 1.18 | -3.96 |  |  |  |  |  |  |
| *qPH9-2* | 9 | 99.75-111.40 | 0.90 | -3.41 |  |  |  |  |  |  |
| *qPH10* | 10 | 82.00-84.95 | 10.89 | 11.11 | 7.85 | 8.71 |  |  |  |  |
| *qEH1-1* | 1 | 90.75-96.90 |  |  | 14.15 | 9.62 |  |  |  |  |
| *qEH1-2* | 1 | 128.95-145.85 | 10.53 | 8.52 |  |  |  |  |  |  |
| *qEH2-1* | 2 | 143.10-162.45 |  |  | 1.93 | -3.55 |  |  |  |  |
| *qEH2-2* | 2 | 204.70-210.25 |  |  |  |  | 3.98 | 0.93 |  |  |
| *qEH4-1* | 4 | 34.15-39.90 | 3.05 | 4.55 |  |  |  |  |  |  |
| *qEH4-2* | 4 | 167.85-199.55 |  |  |  |  | 3.49 | 0.89 | 6.79 | -1.23 |
| *qEH5* | 5 | 172.85-178.10 | 8.77 | 7.70 | 6.19 | 6.32 |  |  |  |  |
| *qEH6-1* | 6 | 30.80-34.50 |  |  | 2.97 | 4.44 |  |  |  |  |
| *qEH7-1* | 7 | 43.65-48.80 |  |  |  |  |  |  | 6.96 | 1.25 |
| *qEH7-2* | 7 | 125.00-129.25 |  |  | 7.80 | 7.15 |  |  |  |  |
| *qEH7-3* | 7 | 174.05-176.65 |  |  |  |  |  |  | 5.32 | 1.08 |
| *qEH8* | 8 | 172.15-175.15 |  |  | 1.99 | 3.59 |  |  |  |  |
| *qEH9-1* | 9 | 11.05-25.90 |  |  | 5.75 | -6.08 | 5.14 | -1.05 |  |  |
| *qEH9-2* | 9 | 129.60-135.05 | 6.53 | -6.61 |  |  |  |  |  |  |
| *qEH10-1* | 10 | 22.70-27.00 |  |  |  |  | 4.01 | 0.95 |  |  |
| *qEH10-2* | 10 | 80.75-86.45 | 12.07 | 9.15 | 6.42 | 6.59 |  |  |  |  |
| *qEH10-3* | 10 | 142.85-145.75 |  |  | 2.02 | 3.75 | 5.15 | -1.05 | 4.22 | 0.96 |
| *qIN1-1* | 1 | 128.95-145.85 | 3.23 | 0.31 |  |  |  |  |  |  |
| *qIN1-2* | 1 | 263.10-268.55 |  |  | 3.33 | -0.30 | 3.83 | 0.12 |  |  |
| *qIN2-1* | 2 | 129.95-153.65 | 5.57 | -0.41 |  |  |  |  |  |  |
| *qIN2-2* | 2 | 194.55-198.25 |  |  | 6.08 | -0.41 |  |  |  |  |
| *qIN3* | 3 | 161.60-175.40 |  |  |  |  | 3.74 | -0.12 |  |  |
| *qIN7* | 7 | 147.45-152.85 | 2.96 | 0.29 |  |  |  |  |  |  |
| *qIN8* | 8 | 83.30-89.35 |  |  | 11.16 | -0.55 |  |  | 3.35 | -0.12 |
| *qIN9* | 9 | 150.65-154.15 |  |  | 4.54 | -0.35 | 3.78 | 0.12 |  |  |
| *qIN10* | 10 | 80.35-86.45 | 9.71 | 0.54 |  |  |  |  |  |  |

^a^*R^2^* represents the phenotypic variation explained by each QTL.

^b^Additive effect of the QTL calculated in the testcross populations. QTL effects were estimated by the heterozygote (Chang7-2 or Mo17/Qi319)—the heterozygote (Chang7-2 or Mo17/Ye478).

The trait dataset abbreviations match those in Figure 1.

PH: plant height; EH: ear height; IN: internode number.
