# Supplementary material for: Dissecting the Genetic Basis Underlying Combining Ability of Plant Height Related Traits in Maize
Source: Front Plant Sci. 2018 Aug 2;9:1117. doi: 10.3389/fpls.2018.01117 (PMC6083371; doi:10.3389/fpls.2018.01117)
Supplement: TABLE S3 — Phenotypic correlation (r) coefficients for plant height related traits in different trait datasets. [file Table_3.DOCX]

Table S3 Phenotypic correlation (*r*) coefficients for plant height related traits in different trait datasets.

| Trait | RIL | TC | TM | GCA | SC | SM |
| --- | --- | --- | --- | --- | --- | --- |
| PH vs EH | 0.74** | 0.78** | 0.76** | 0.79** | 0.60** | 0.57** |
| PH vs IN | 0.36** | 0.42** | 0.37** | 0.41** | 0.63** | 0.64** |
| EH vs IN | 0.51** | 0.40** | 0.34** | 0.42** | 0.34** | 0.40** |

**P* < 0.05; ***P* < 0.01

PH: plant height; EH: ear height; IN: internode number.

The trait datasets abbreviations match those in Fig. 1
