# Supplementary material for: Dissecting the Genetic Basis Underlying Combining Ability of Plant Height Related Traits in Maize
Source: Front Plant Sci. 2018 Aug 2;9:1117. doi: 10.3389/fpls.2018.01117 (PMC6083371; doi:10.3389/fpls.2018.01117)
Supplement: TABLE S5 — Pleiotropic QTL identified in different combinations for plant height related traits. [file Table_4.DOCX]

Table S4 Pleiotropic QTL identified in different datasets for plant height related traits.

| Chr. | Interval (Mb) | RIL | GCA | TC | TM | SC | SM |
| --- | --- | --- | --- | --- | --- | --- | --- |
| 1 | 128-157 | EH/IN | PH/EH | PH/EH/IN | PH |  |  |
|  | 251-268 |  |  | PH | PH/IN | PH/IN |  |
| 2 | 129-153 |  | IN | IN | EH |  |  |
|  | 198-210 | PH |  |  | IN | EH |  |
| 3 | 153-165 | EH |  |  |  | IN | PH |
|  | 190-208 | PH/IN |  |  |  |  |  |
| 4 | 177-197 |  | PH | PH |  | EH | PH/EH |
| 5 | 171-190 | PH/EH/IN | PH/EH | PH/EH | PH/EH | IN |  |
| 6 | 93-107 | PH/EH |  |  |  |  | PH |
|  | 157-164 |  | PH | PH |  |  |  |
| 7 | 25-46 |  |  |  |  | EH | EH |
|  | 125-131 |  | PH/EH |  | PH/EH |  |  |
|  | 162-174 |  | IN | PH |  | PH | EH |
| 8 | 72-89 |  | PH/IN | PH | IN |  | IN |
|  | 154-172 | PH |  |  | PH |  | PH |
| 9 | 9-23 |  | PH/EH | PH | EH | EH |  |
|  | 131-154 | EH |  | EH | IN | IN |  |
| 10 | 82-88 |  | PH/EH/IN | PH/EH/IN | PH/EH |  |  |
|  | 144-147 |  |  |  | EH | EH | EH |

The trait dataset abbreviations match those in Figure 1.

PH: plant height; EH: ear height; IN: internode number.
